# Does network homophily persist in multicultural volunteering programs? Results from an Exponential Random Graph Model

**DOI:** 10.1016/j.jmh.2024.100256

**Published:** 2024-07-28

**Authors:** Qiuchang (Katy) Cao, Holly Dabelko-Schoeny, Keith Warren, Mo Yee Lee

**Affiliations:** a Florida State University College of Social Work, Tallahassee, FL 32304, United States; b The Ohio State University College of Social Work, 1947 College Road North, Columbus, OH 43210, United States

**Keywords:** Ethnic/racial minorities, Immigrant/refugee, Social network analysis, Volunteering, Older adults

## Abstract

Few studies examined the social network structures within multicultural volunteer programs for low-income diverse older adults, making it unclear how diverse older adults establish social connections beyond their co-ethnic community. This study aims to identify the social network structures within a Senior Companion Program (SCP), a multicultural low-income volunteer program in a Midwestern Metropolitan area in the United States. Data were collected through surveys during a SCP monthly in-service training in October 2021. Russian, Khmer, Somali, Nepali, and English-speaking older volunteers in the SCP (*N* = 41) identified friends through a nomination form. Exponential Random Graph Modeling (ERGM) was used to identify statistically significant structural features of the SCP network. Graphs and ERGM results demonstrated that participants tended to form friendships with other volunteers of the same gender (β=3.27, *p* < 0.001), from the same country (β=2.89, *p* < 0.001), with the same education level (β=0.71, *p* < 0.001), and from the same volunteer recruitment site (β=2.77, *p* < 0.001). Surprisingly, there were few transitive ties (β= -1.01, *p* < 0.001), the tendency to make friends with a friend of a friend, which is typically common in friendship networks. Relationships among diverse older volunteers are largely driven by homophily in this multicultural volunteer program. Addressing language barriers and assigning volunteers from different countries to the same recruitment site may counteract homophily by nationality. However, more research needs to identify whether the opportunity to interact with people of one's *same* or *different* cultural backgrounds is a stronger incentive for volunteer engagement and connectedness.

## Introduction

The social networks among immigrants and refugees are frequently characterized by network closure and homophily (similarity)-driven relationships with tight-knit kinship networks and co-ethnic communities (e.g., ethnic enclaves, ethnoburbs) in the existing literature (Fukui and Menjívar, 2015; Li, 2018; Torres and Serrat, 2019; Vacca et al., 2022). Some scholars refer to this *homophily-based* socialization pattern as ethnic solidarity whereas others interpret it as segregation due to structural exclusion and cultural preferences (Vacca et al., 2022). However, recent studies have questioned the prevailing assumption that homophily is the defining characteristic of migrants’ social networks (Becker, 2022; Vacca et al., 2022). For instance, Romanian immigrants in France established extensive networks beyond their co-ethnic community to meet different needs (e.g., financial, professional, housing), seek varying opportunities, and enhance upward social mobility (Vacca et al., 2022). Their network structures mimicked the ethnic majority in France, reflecting networked individualism, characterized by sparse and weak ties connecting heterogeneous social circles and resources to meet an individual's various needs (Vacca et al., 2022). Although some studies have suggested that older immigrants tend to rely more on co-ethnic communities than their younger counterparts (Szabó et al., 2023), there is little information on diverse older adults’ full social network structure in settings beyond their co-ethnic communities.

The lack of research on multicultural interactions among diverse older adults stands in stark contrast to the extensive scholarship on their kinship networks and engagement in co-ethnic communities (e.g., Ciobanu et al., 2017; Li and Dong, 2018; Viruell-Fuentes et al., 2012, 2013; Wright-St Clair et al., 2018). Multicultural volunteering programs provide unique opportunities for network expansion beyond one's kinship and co-ethnic community among older immigrants and refugees (Wiles et al., 2019). However, the social dynamics within multicultural volunteer programs remain unclear. The unknown social patterns within multicultural volunteer programs raise questions about whether promoting such initiatives is a worthwhile means of building social connections, especially when fewer linguistic and cultural barriers exist for diverse older adults to engage in their co-ethnic communities informally (e.g., Wright-St Clair et al., 2018). To shed light on diverse older adults’ social interactions beyond their co-ethnic community, this study examines how diverse older adults in a multicultural volunteer program, the Senior Companion Program (SCP) in a Midwest metropolitan area, are connected.

Senior Companion Program (SCP) is a means-tested (determining individuals’ eligibility for benefits based on their income) federal volunteer program in the United States that recruits low-income (at or below 200 % of the federal poverty line) older adults who are 55 years and older (volunteers) to provide support and companionship for homebound older adults (clients) (Tan et al., 2016). Compared with volunteer programs that are not means tested, volunteers of SCP are more likely to be racially and ethnically diverse (Hood et al., 2018). The local SCP featured in this study is in a Midwest metropolitan area with a growing number of minoritized immigrants and refugees. Through the partnership with local nonprofit organizations that serve immigrants/refugees and older adults (e.g., Community Refugee and Immigrant Services [CRIS], Asian American Community Services [AACS], Senior Options) referred to as recruitment sites, the SCP recruited a group of culturally and linguistically diverse older volunteers and clients into the program via talks in the community. Once recruited, older volunteers are managed and supervised within their recruitment sites. Besides connecting with clients based on similar ethnicity, preferred language, cultural background, and shared interest, volunteers in the SCP also have regular opportunities to interact with other volunteers from various cultural backgrounds through organized group opportunities (Tan et al., 2016), such as volunteer orientation, monthly in-service training, and volunteer recognition events.

## Theoretical framework

Social network theory (SNT) provides conceptual tools for examining how structures within social networks (e.g., clustering, network density) impact individuals’ attitudes, behaviors, health, and overall well-being, in conjunction with factors external to the network processes (e.g., sociodemographic characteristics) (Borgatti et al., 2013; Valente and Pitts, 2017). According to SNT, people tend to form friendships with those who *reciprocate* positive interactions (Borgatti et al., 2013; Kadushin, 2012). Early in the life course, humans learn to reciprocate as an important means to develop and maintain friendships (Kadushin, 2012). Identifying the significance of reciprocity in SCP sheds light on the depth of relationships within the multicultural program.

Furthermore, individuals are more likely to establish relationships if they share a common friend (*transitivity*) (Borgatti et al., 2013; Kadushin, 2012). Unlike physical or biological networks (such as neural connections), human social networks uniquely exhibit transitivity (Kadushin, 2012). Transitivity in this study reflects older volunteers' embeddedness within SCP.

Similarities also nurture and reinforce social connections, a social network feature referred to as *homophily* (Valente and Pitts, 2017). Researchers disagree over the costs and benefits of homophily. Homophily is sometimes considered a barrier to the social integration of marginalized groups and can negatively impact their employment and economic opportunities (Henry, 2021; Paolillo and Lorenz, 2018). Other researchers argue that homophily fosters a sense of safety and belongingness within the group, which is a basic psychological motivation for establishing social relationships (Kadushin, 2012; Q. Li, 2018). Building upon the aforementioned debate between *homophily* versus *networked individualism* in diverse communities, we investigate whether homophily remains prevalent in this multicultural program where opportunities for cross-cultural interactions are available.

Informed by SNT, social network analysis (SNA) undertakes the statistical examination of social interactions within a network (Borgatti et al., 2013). The focus on patterns of social interactions differentiates SNAs from traditional studies on social support and studies reporting aggregated measures of a network (e.g., network size, network composition) (Cornwell et al., 2008; Schafer et al., 2018). SNAs have different purposes and can be divided into two categories: (1) ego/personal network analysis which focuses on individuals’ immediate social network (e.g., Ortiz et al., 2021; Ruiz et al., 2022), and (2) whole network analysis which investigates the interactions among everyone within a defined network (Borgatti et al., 2013).

Ego/personal network studies generally focus on how each individual's immediate social contacts (e.g., families, co-ethnic social contacts) shape one's access to resources and support (Fernández-Peña et al., 2018; Small et al., 2021). However, the individual focus in ego/personal network analysis provides limited information on the complex patterns of interactions among various members within a social network. Taking a broader perspective, whole network analysis collects information on the social interactions among all available network members, usually within a certain boundary/context for feasibility (Liu et al., 2017). Whole network studies are appropriate for studying patterns and dynamics of interactions among individuals in certain social settings (e.g., a program or organization) (Kadushin, 2004; Liu et al., 2017). To inform future multicultural programming beyond co-ethnic communities, this study takes advantage of whole network analysis to understand diverse older adults’ patterns of social interaction in SCP.

### Social network analysis and older adults

In contrast with the growing interest in older adults’ ego/personal networks, whole network studies among community-living older adults are scarce in the gerontological literature (Ayalon and Levkovich, 2019). According to a systematic review, the few existing whole network studies in gerontology focus mainly on middle-class white older adults in long-term care facilities (e.g., nursing homes, assisted living facilities, memory care units, or continuing care facilities) or retirement communities that have clear network boundaries (Ayalon and Levkovich, 2019). These whole network studies suggested that older adults’ social networks were low in density and reciprocity (Ayalon and Levkovich, 2019). Additionally, organizational structures and contexts shaped the network structures of older adults (Casey et al., 2016; Hardiman, 2017; Mauldin et al., 2021; Schafer, 2015, 2016). For instance, older residents of long-term care facilities were more likely to establish friendships with those who were on the same floor (proximity) and entered the facility at approximately the same time (Casey et al., 2016; Mauldin et al., 2021; Schafer, 2015).

### Volunteering and older adults’ social networks

Consistent with the lack of whole network studies in gerontology (Ayalon and Levkovich, 2019), few studies have examined the whole social network within volunteer programs for older adults. Existing SNAs on older volunteers mostly focused on the association between their ego/personal networks outside of the volunteer program and volunteer participation (e.g., Dávila, 2018; Pilkington et al., 2012; Webster et al., 2021). For example, older adults with larger social networks and better social support are more likely to engage in volunteering (Dávila, 2018; Pilkington et al., 2012). Participating in volunteering is further positively associated with increased personal network sizes, especially among older adults who experienced social losses (Jiang et al., 2019; Pilkington et al., 2012; Webster et al., 2021). According to previous descriptive SNA on SCP volunteers (Cao et al., 2023), participants in this multicultural program interacted mostly with those from the same country, sharing the same race, and gender identities. However, inferential SNA is needed to further identify which network structure is significant for forming social relationships in this local SCP.

Moreover, previous research on culturally and linguistically diverse older adults’ community engagement has primarily centered around their informal volunteering within their co-ethnic communities, which tend to be racially or ethnically homogenous (e.g., Wright-St Clair et al., 2018, Torres and Serrat, 2019). Although scholars advocated for the inclusion of older immigrants and refugees in organized volunteering programs (e.g., Gonzales and Nowell, 2017; Torres and Serrat, 2019), we are not aware of any inferential SNA examining the patterns of social interaction within multicultural volunteering programs. Understanding how diverse older adults interact within SCP informs future research on the benefits of multicultural volunteering programs in addition to co-ethnic communities. These insights can also guide practitioners in shaping social relationships within multicultural programs based on programmatic goals. Furthermore, moving beyond personal networks at the individual level, the whole SNA of a multicultural volunteering program provides a snapshot of how individual identities, organizational policies, and broader social contexts intersect to structure diverse older adults' social interactions.

## The current study

To address the unanswered questions in existing personal network analysis among different groups of older adults, this inferential whole network moves beyond their direct social circle, shedding light on the social dynamics and patterns among members within a multicultural volunteer program. Specifically, we addressed the following research questions: What are the characteristics of the friendship network among diverse older volunteers of SCP? Specifically, we examine (1) Is friendship reciprocated? (2) Is it transitive? (3) Does homophily by age, gender, race, country of origin, race, education, and recruitment site facilitate friendships in SCP? Informed by the typical network structures in friendship networks according to SNT (Borgatti et al., 2013; Kadushin, 2012), we hypothesize that all homophily variables, reciprocity, and transitivity are positively associated with friendship tie formation within SCP.

## Methods

### Sampling and participants

This study utilized convenience sampling of all current volunteers in the SCP in a Midwest metropolitan area. All SCP volunteers in the local program were eligible for this study. Because of COVID-related restrictions and concerns, a total of 41 volunteers took the social network survey. In addition to nominating others who also took the network survey as friends within SCP, participants nominated 42 friends who were not present during data collection. The inclusion of those who did not participate would have induced a bias against reciprocity in the network because it is impossible for a non-participant to reciprocate a friendship nomination. To avoid this bias, the analysis only included friendship nominations among participants who were present at the data collection, resulting in a sample size of *N* = 41 for this inferential SNA. The Institutional Review Board (IRB) of the Ohio State University (Study ID: 2021B0254.) reviewed and approved the study procedures.

### Data collection

Participants completed a sociodemographic survey and a network nomination form in group settings during the SCP monthly in-service training in October 2021. All participants consented to data collection. After providing their demographic information, participants completed the friendship nomination form in the language they preferred (English, Nepali, Somali, Khmer, and Russian). Each participant (ego) was asked to identify the names of five friends (alters) they met through the Senior Companion Program (SCP), their relationship with each alter, and the alters’ characteristics (e.g., age, gender). Using a name roster of volunteers provided by SCP, the facilitators asked participants to refer to the name roster and provide the names of their friends in English whenever possible. Details regarding study participants, sampling and recruitment, and data collection were reported in the previous manuscript (Cao et al., 2023).

### Measures

Age (numeric), gender (male, female), country of origin (United States, China, Bhutan, Cambodia, Nepal, Russia, Somalia, and other), race (White, Black or African American, American Indian or Alaska Native, Asian or Pacific Islander, Hispanic or Latino/Latina/Latinx, and other), and highest level of education (no high school degree, high school degree or equivalent, some college, no degree, Associate degree, Bachelor's degree, and graduate or professional degree) were collected through participants’ self-report in the social demographic survey. In addition, participants were asked to share their age of migration and length of residence in the U.S. if they were not born in the U.S.

When an ego nominated an alter in the friendship nomination form, a directional tie was defined from ego to alter. The strength of the tie was then evaluated through the question: “How many times have you interacted with (e.g., in-person, phone) this person in the past month?” The frequency of interactions as reported by volunteers was then introduced as the network weight for the valued Exponential Random Graph Models (ERGM) in this study, which is explained in the Analysis section. Modeled structures in the friendship network included reciprocity and transitivity, as presented in Appendix I. The friendship nomination form also allowed egos to share the demographic characteristics (age, gender, race, country of origin, highest level of education) of alters based on egos’ knowledge. After obtaining the characteristics of egos and alters, the homophily of a numeric variable (i.e., age) was calculated as the absolute difference between the values of two nodes whereas the homophily of categorical variables was calculated by whether or not two connected nodes/individuals shared the same attribute (e.g., male, female) (Cranmer et al., 2020).

### Data analysis

Network visualization and descriptive analysis (Borgatti et al., 2009, 2013) were conducted to uncover social structures in SCP. Furthermore, inferential network analysis using the Exponential Random Graph Model (ERGM) was calculated to answer our research question regarding statistically significant structures associated with relationship formation and dissolution within a network (Wasserman and Pattison, 1996). ERGM identifies factors endogenous (e.g., transitivity, reciprocity) and exogenous (homophily) to the network that are statistically significant to the formation of relationships (Cranmer et al., 2020). Through modeling various networks ranging from completely empty (no connections among nodes) to completely connected (all nodes connected) based on the fixed set of nodes, ERGM predicts the possibility of observing the current network over all possible networks that share the same number of nodes. ERGM uses the Markov Chain Monte Carlo (MCMC) methodology to simulate the networks (Cranmer et al., 2020).

ERGM has two major advantages when applied to network data. First, ERGM models network structures that would otherwise be ignored in traditional regression analysis (Cranmer et al., 2020). Modeling the whole network enables ERGM to account for factors endogenous and exogenous to the network when predicting the probability of tie formation. Factors endogenous to a network usually include edge-level properties, such as the possibility of people preferring to interact with popular peers or preferring to interact with those that reciprocate (Cranmer et al., 2020). Factors external or exogenous to a network usually include node-level properties, such as participants’ characteristics (e.g., age, race, and gender) (Cranmer et al., 2020). Additionally, unlike regression analysis which assumes independence of observations, ERGM accounts for autocorrelation in the network data (Cranmer et al., 2020). Observations of a network structure are often dependent upon one another. For instance, the friendship tie between person A and person B cannot be reciprocal unless both A and B nominate each other as a friend. Because ERGM treats the entire network as a single observation, it avoids making independent and identical distribution (i.i.d) assumptions regarding the observations (Cranmer et al., 2020).

ERGM algorithms are suitable for a wide range of network sizes, node covariates, and edge properties (Cranmer et al., 2020). The statistical assumptions of ERGMs are minimal. The primary assumption of ERGM is that the probability of observing two networks with the same values on the selected statistics should be the same (Cranmer et al., 2020). The addition of exogenous or endogenous factors can increase the chance of observing a certain network over the other. The second assumption of ERGM expects any sample of networks from the fitted distribution to have their network statistics centered around the observed network (Cranmer et al., 2020). In other words, the observed network statistics should be representative of the population statistics in order to generalize. The Markov Chain Monte Carlo Maximum Likelihood Estimation (MCMC-MLE) produces goodness of fit statistics to evaluate ERGMs. Models violating the statistical assumptions of ERGM show poor model fit (Cranmer et al., 2020).

The concept of statistical power in ERGM is different from power in regression-type analyses (Krivitsky and Kolaczyk, 2015). Statistical power in regression-type analyses typically depends on the size of the sample that is collected from the field. However, in ERGM, each simulated network is a data point (Krivitsky and Kolaczyk, 2015). Statistical power depended on being able to simulate networks in a substantial region of the space of networks given the fixed set of nodes in the observed network (Cranmer et al., 2020). Like most other MCMC applications, the simulated sample in ERGM consisted of a chain of simulated networks, where each network is a slight variation of the previous one. The ERGM package for R automated the process of finding an appropriate burn-in, thinning interval, and effective sample size, enabling the lead researcher to specify a desired precision for the statistical inference and the desired level of trustworthiness of parameter estimates and their significance scores (Krivitsky and Kolaczyk, 2015). The current state of the art in SNAs is to evaluate the goodness of fit of models after model specification and determine if the sample was sufficient for the model after data collection (Krivitsky and Kolaczyk, 2015). Good model fit indicates trustworthy parameter estimates (Cranmer et al., 2020). The statistical degeneracy in ERGM and the count ERGM implemented for this study are further explained in Appendix II. To enhance the transparency and reproducibility of this study, we presented our data (edge list and node attribute) as well as code for analysis and model fit evaluation in the supplementary material.

## Results

### Demographic characteristics

Table 1 displays the demographic characteristics of the 41 volunteers who participated in the data collection. The mean age of participants was 76.99 (*SD*=9.09). Approximately 52.63 % of the sample was female. Older volunteers in the program identified with various countries of origin. For instance, 39.02 % of participants were from the USA, 24.39 % were from Russia, 12.20 % were from Cambodia, 7.32 % were from Bhutan or Ukraine, and 4.88 % were from Somalia. In terms of race and ethnicity, 51.22 % of participants identified as White, 26.83 % were Black or African American, and 19.51 % identified as Asians or Pacific Islanders. On average, older migrants in this sample spent 26.12 years in the US (*SD*=11.73) and migrated at an average of 52.04 years old (*SD*=13.14). Approximately 56.52 % of older migrants in the sample were refugees or asylees.

**Table 1:** Descriptive node statistics.

Variable	Frequency	%	Mean	SD	*N*
Age			76.99	9.09	41
Gender					38
Female	20	52.63			
Male	18	47.37			
Country of origin					41
Bhutan	3	7.32			
Cambodia	5	12.2			
Ethiopia	1	2.44			
German	1	2.44			
Russia	10	24.39			
Somalia	2	4.88			
Ukraine	3	7.32			
USA	16	39.02			
Race					41
Asian or Pacific Islander	8	19.51			
Black or African American	11	26.83			
White	21	51.22			
Other	1	2.44			
Education					37
No high school degree	8	21.62			
High school degree or equivalent	6	16.22			
Some college no degree	8	21.62			
Associates degree	2	5.41			
Bachelor's degree	5	13.51			
Graduate or professional degree	8	21.62			
Employment					40
Employed part-time	3	7.5			
Retired and not looking for work	27	67.5			
Self-employed	1	2.5			
Unemployed but looking for work	3	7.5			
Other	6	15			
Marital status					40
Divorced or separated	5	12.5			
Married	19	47.5			
Never married	5	12.5			
Widowed	11	27.5			
Household composition					39
Live alone	17	43.59			
Live with spouse	19	48.72			
Live with children	6	15.38			
Live with grandchildren	2	5.13			
Live with other relatives	1	2.56			
Years of residence			26.12	11.73	24
Migration age			52.04	13.14	23

*Note*. The number of family members refers to the number of family members seen or heard from at least once a month. The number of friends outside of SCP refers to the number of friends seen or heard from at least once a month. Only participants who were not born in the U.S. were instructed to respond to questions regarding their years of residence in the U.S., their age of migration, and reasons for migration.

When considering the nature of the relationship between egos and alters, around 80.65 % of the participants nominated another volunteer of SCP as a friend. Additionally, 61.9 % of participants introduced the friends they met at SCP to friends and family members outside of SCP, indicating that these relationships transcended program boundaries. However, the average frequency of interaction between the ego and the alter in the past month was relatively low (*Mean*=3.22), with an *SD* of 4.98. It is possible that COVID-19 restrictions at the time of data collection limited the frequency of interactions among volunteers. Appendix III illustrates the descriptive characteristics of edges (ties) in the network.

### Network descriptive information and graphs

Network graphs of participants in the data collection include a large number of isolates (i.e., people with no ties). From Appendix IV to VIII, isolates are arranged on the right side of each network graph. Some of these isolates had friends in the program who were not present when data was collected. Separate network graphs were constructed to visualize different types of homophily or heterophily based on gender, education, country of origin, race, and recruitment sites within SCP. The size of each node in the network graph reflected the total degree (indegree plus outdegree) of each ego (Freeman, 1979). Total degree is a more accurate centrality measure than indegree because directional measures of centrality (e.g., in-degree, out-degree) are more likely to be influenced by missing data. The larger the node, the higher total degree it had. The thickness of the arrows represented the frequency of interactions in the past month. A thicker arrow represented a higher frequency of meetings. The direction of the arrows represents who nominated whom.

Females and males are represented in different colors in Appendix IV. No participants of this study selected “other” as gender in the survey. Appendix IV illustrates four gender homophilous clusters and two with mixed gender (male and female). Comparing participants’ gender among various focus groups (as indicated by their IDs), the graphs also illustrate that all nodes in the Nepali-speaking and Somali-speaking groups were male whereas the majority of nodes in English-speaking groups were female.

Different levels of education are presented in different colors in Appendix V. Clusters of individuals with the same level of education can be identified in the network with some exceptions. Furthermore, the highest level of education varied among different language groups. For instance, all nodes in the Nepali-speaking groups had no high-school degree whereas the majority of nodes in the Russian-speaking groups had graduate or professional degrees.

In Appendix VI, each country is represented by a color. As indicated by clusters with the same color, the SCP network displayed a clear tendency of homophily based on country according to the network graph. Similar to other network graphs, there is no tie across clusters. Participants tended to nominate people from the same country as friends.

In Appendix VII, each color represented a racial category. The majority of clusters demonstrated race-based homophily with two exceptions. In Appendix VIII, each color represents a recruitment site of SCP. Cambodian participants were all from AACS, whereas all Bhutanese and Somali participants were from CRIS. Cambodian participants formed a homophilous cluster based on their recruitment sites. Although both Bhutanese and Somali participants were served by CRIS, there was no cross-over between the two clusters in the graph. English-speaking participants also formed recruitment site-based homophily with two exceptions.

### Exponential Random Graph Models (ERGM)

Results from the count ERGM are presented in Table 2. Except for age and race, all homophily variables were positively significant in this network. In other words, volunteers were more likely to be friends with those from the same country, of the same gender, with the same educational level, and from the same recruitment site. Although nationality-based homophily was significant, race-based homophily became non-significant once recruitment site-based homophily was introduced. The VIFs of all independent variables (e.g., homophily based on country, race, and recruitment sites) were below 20 in the final ERGM, suggesting that multicollinearity did not unduly influence the results (Duxbury, 2021).

**Table 2:** Results from the valued ERGM.

	Estimate	Std. Error	z value	Pr(>|z|)	
Sum of the frequency of meetings	−8.8	0.73	−11.96	< 1e-04	***
Homophily by country of origin	2.89	0.44	6.5	< 1e-04	***
Homophily by gender	3.27	0.55	6	< 1e-04	***
Homophily by education	0.71	0.21	3.37	0.0007	***
Homophily by recruitment sites	2.77	0.31	8.94	< 1e-04	***
Transitive ties	−1.01	0.27	−3.69	0.0002	***
Homophily by race	0.03	0.22	0.14	0.89	
Homophily by age	0.02	0.02	1.31	0.19	
Reciprocity	−0.1	0.31	−0.33	0.74	

Note. p***<0.001***; p**<0.01, p*<0.05, p^.^<0.1. AIC: −2971; BIC: −2922.

Although reciprocity could be observed visually in network graphs, it was not statistically significant in ERGM. Additionally, the negative significance of transitive ties suggested that there was a tendency against forming transitive triads within this network. That is to say, participant A who nominated participant B as a friend was unlikely to have a friend who also nominated B.

Variables were added to ERGM one by one, and the final model had the smallest Akaike information criterion (AIC) and Bayesian Information Criteria (BIC), indicating a better fit for the model (Cranmer et al., 2020). The final model had an MCMC sample size of 524,288 and the MCMC burn-in was 30,720. A large MCMC sample size and long MCMC burn-in facilitated model convergence. The convergence of each model parameter was also illustrated by the non-trending MCMC trace plots (Cranmer et al., 2020) displayed in Appendix IX.

**Fig. 1:**
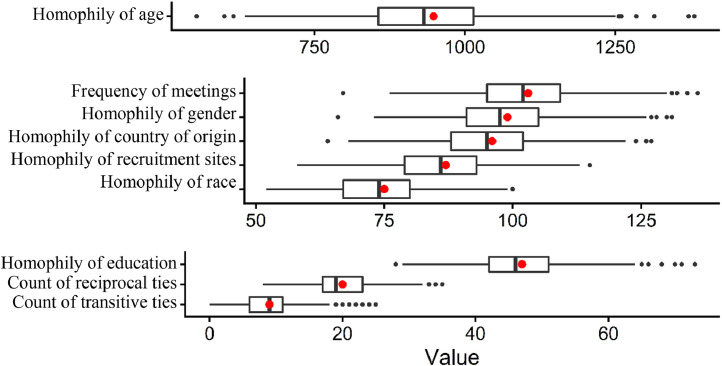
The Goodness of Fit Plot *Note.* The boxplot represented a random sample of 1000 simulated networks using the parameter estimates from Table 2. The networks were simulated from the exponential probability distribution with the same Poisson reference used in the ERGM fit. The simulated networks also had the same number of nodes as the observed network. Unless otherwise noted, all amounts shown are total sums across edges.

Aside from the MCMC trace plots, another set of tools for assessing the fit of ERGM is the goodness of fit statistics and goodness of fit plot, which are currently unavailable for count-ERGM in R. However, the lead researcher was able to construct the goodness of fit plots (boxplots) by comparing the characteristics of simulated networks with the observed network visually (Cranmer et al., 2020). In Fig. 1, the box plots summarized the properties (e.g., homophily, transitive ties) of the 1000 simulated networks, whereas the red dots in Fig. 1 represented the corresponding property in the observed network. The proximity between each red dot and the medians of the boxplots indicates that ERGM successfully simulated the properties of the observed network, reflecting a good model fit (Cranmer et al., 2020).

## Discussion

As the first inferential SNA on a multicultural volunteering program to our knowledge, this study revealed network dynamics associated with friendship formation among volunteers within one SCP. The hypotheses that all homophily variables, reciprocity, and transitivity were positively associated with relationship formation within SCP were only partially supported by the results from ERGM. Consistent with the existing studies on diverse older adults’ social interactions within their co-ethnic community (Li, 2018; Torres and Serrat, 2019), homophily based on country of origin, gender, education, and recruitment site was positively associated with tie formation in this multicultural program. Recruiting and managing volunteers from homophily-based recruitment sites further strengthened nationality-based homophily within SCP. However, the endogenous network structures (i.e., transitivity and reciprocity) did not positively contribute to the tie formation as commonly seen in friendship networks (Borgatti et al., 2013). Because reciprocity is common in friendship ties (Borgatti et al., 2013), the non-significance of reciprocity in this network suggests that alters who were nominated as friends within SCP might not be considered friends beyond the program.

The findings of this exploratory study, therefore, imply that program managers should be aware that the meaning of the term “friendship” appears to be contextual, with the term being applied somewhat more casually in a volunteer setting. In this SCP, although volunteers may be motivated by the presence of people similar to themselves, ERGM results indicated that there were few deep or reciprocal friendships in the network. In other words, friends in the volunteer program might be similar to “work friends” with whom people do not necessarily interact outside of their workplace.

The lack of deep friendships in SCP (indicated by few strong ties and limited reciprocity) may also explain the negative value of transitivity in the ERGM. Transitivity occurs because people want to preserve friendships (Hummon and Doreian, 2003). If the typical friendship is not particularly deep in SCP, it is reasonable to see fewer transitive triads within a network. In this study, contextual factors such as the volunteering format in SCP (i.e., one-on-one companionship visits) and the priority of forming relationships with clients are comparatively salient, potentially contributing to the avoidance of forming transitive triads with other volunteers in the network.

Findings from this study supported the *network homophily* argument (Fukui & Menjívar, 2015; Li, 2018) rather than *networked individualism* in the literature (Becker, 2022; Vacca et al., 2022). The opportunity to bond with other volunteers from the same country, of the same gender (Tan et al., 2016), with the same level of education, and from the same recruitment site sustains diverse older adults’ ongoing participation in SCP. This is likely because our study featured low-income diverse older adults in the U.S. with distinct pre- and post-migration conditions compared with migrants in Europe (Becker, 2022; Vacca et al., 2022). The discrepancy also reflects the methodological differences between personal network analysis (Becker, 2022; Vacca et al., 2022) and this whole network analysis. Considering the heterogeneity within the migrant community, both ego-network and whole network studies are needed to comprehensively understand different groups of older migrants’ social interactions beyond their ethnic enclaves/ethnoburb in the U.S. Additionally, race-based homophily became non-significant after site station-based homophily was introduced. The overlap between site stations and race/ethnicity in SCP might have explained why race-based homophily became non-significant once site station-based homophily was added to the model. In other words, what appeared to be race-based homophily might have been site-based homophily in this setting. In contrast, nationality-based homophily remained significant even with the inclusion of site stations in this study, implying that nationality might be more relevant than race in understanding diverse older adults’ experiences in this multicultural program. However, more studies need to examine whether and how first-generation older migrants from developing countries experience racialization in the U.S. and whether racial categories impact their self-perceived identities.

Moreover, building on the importance of organizational facilitation (e.g., incentives and training) in recruiting and retaining diverse older adults in existing literature (Gonzales et al., 2015; Hong and Morrow-Howell, 2013), the significance of recruitment site homophily in this network suggests that the organizational structure of volunteer programs can strengthen or weaken homophily. Because homophily in social relationships is usually a result of individual choice and environmental/structural constraints (Ertug et al., 2022; Firmansyah and Pratama, 2020), individuals may connect with similar peers out of personal preference or may do so due to external barriers/facilitators for forming cross-cultural relationships (Ertug et al., 2022; Melamed et al., 2020). Intensifying or relaxing site-based recruitment and management of volunteers can also have an impact on the overall homophily of relationships within SCP.

### Limitations

This study can be regarded as a cross-sectional case study of one SCP network and thus has limited generalizability to other SCPs or volunteer programs. Findings do not offer causal explanations for volunteer participation and relationship formation. Additionally, due to the impact of COVID-19, only approximately half of SCP volunteers participated in the data collection, contributing to the missing data in the friendship nomination form. The missing data in this network might have also influenced the statistical significance of network structures such as transitivity and reciprocity when running ERGM (Green et al., 2019). However, other studies demonstrated that transitivity is robust to missing data in SNA (Gile and Handcock, 2017). Moreover, older volunteers who participated in the data collection and those who did not might have differed in critical sociodemographic characteristics, such as health/functional status and social support. Thus, future studies should further examine the networks of SCPs and other volunteer programs for older adults to understand how relationships form in various volunteer contexts.

### Implications

#### Research

This study demonstrated that ERGM can effectively identify statistically significant structures that are correlated with relationship formation within a network of diverse older adults. Building on previous ERGM studies on institutionalized older adults (e.g., Mauldin et al., 2021), more gerontological studies should consider applying ERGM to reveal the often-overlooked social structures/patterns within programs serving community-living older adults, such as volunteer programs. Considering the prominence of homophily within the SCP network, future network studies should examine the causes and outcomes of the different types of homophily among diverse older adults in multicultural volunteer programs. Understanding how societal, structural, organizational, interpersonal, and individual preferences contribute to homophily in social relationships and investigating the health and well-being outcomes associated with different types of homophily can shed more light on when homophily needs to be encouraged or interrupted. More longitudinal network studies are needed to examine the relationship between volunteering and the volunteers’ social network change over time to better comprehend the social benefits of volunteering.

#### Practice

In multicultural volunteer programs for diverse older adults like SCP, nationality-based homophily naturally fosters connections, bypasses language and cultural differences encountered in cross-cultural relationships, provides incentives for recruiting and retaining diverse older adults, and eases volunteer management. In other words, social interactions within an organized multicultural program did not interrupt homophily-based relationships (Reyes, 2022). Given the role of recruitment sites in strengthening relationship homophily among older adults, it may be possible to alter homophily using organizational structures. If the expansion of social ties and exchange of resources across groups were to be the goal, SCP can consider initiating conversations among recruitment site supervisors to coordinate complementary resources that may enhance diverse older volunteers’ access to a broader range of services (e.g., transportation, health) and opportunities (e.g., employment) across sites to foster weak ties among different groups of diverse older adults. Furthermore, program managers and volunteer coordinators should also be aware that the social networks that diverse volunteers form may serve different functions from typical friendships, even when volunteers describe peers as friends. Findings also suggested that merely providing opportunities for cross-cultural interaction might be insufficient in challenging homophily-based societal structures. If fostering cross-cultural friendship were to be a programmatic goal, regular interpretation and translation services might be needed to foster communication across language groups. Intentional and culturally appropriate socialization opportunities for people of different education, race, and gender can also facilitate cross-cultural interactions. In other words, fostering cross-cultural relationships among volunteers requires additional programmatic interest and commitment.

## Declaration of generative AI and AI-assisted technologies in the writing process

During the preparation of this work, the authors used Microsoft Copilot in order to improve the readability of the language. After using this tool/service, the authors have reviewed and edited the content as needed and take full responsibility for the content of the publication.

## Ethical approval

The study procedures were reviewed and approved by the Institutional Review Board (IRB) of the Ohio State University in the United States, Study ID 2021B0254.

## CRediT authorship contribution statement

**Qiuchang (Katy) Cao:** Conceptualization, Methodology, Data curation, Software, Validation, Formal analysis, Investigation, Resources, Project administration, Visualization, Writing – original draft, Funding acquisition. **Holly Dabelko-Schoeny:** Writing – review & editing, Resources, Project administration, Investigation. **Keith Warren:** Writing – review & editing, Validation, Methodology. **Mo Yee Lee:** Writing – review & editing, Resources.

## Declaration of competing interest

The authors declare that they have no known competing financial interests or personal relationships that could have appeared to influence the work reported in this paper.

## Figures and Tables

**Table:** 

Endogenous Effect	Diagram	Definition
Reciprocity		The count of mutual pairs in a network. A pair of nodes i and j is said to be mutual if both ties i→j and j→i exist.
Transitivity		A transitive triple is defined as a set of 3 nodes i, k_1_, j, together with ties i→k_1_, k_1_→j, i→j. ERGM term *ttriple* counts 2 triangles in this diagram, which is prone to degeneracy.
Transitive ties		A tie i→k is called transitive if at least one two-path i→j→k exists; or, equivalently, if an alter j exists such that both i and j agree in nominating k. ERGM term *transitiveties* counts only 1 tie in this diagram and the one above it; it is less prone to degeneracy.

**Table:** 

Variable	Frequency	%	Mean	SD	*N*
Introduced alter to families/friends outside of SCP					21
No	8	38.1			
Yes	13	61.9			
How the ego first met the alter					31
This alter is a client from the SCP	2	6.45			
This alter is also a volunteer of the SCP	25	80.65			
Other	4	12.9			
Meetings			3.22	4.98	32

*Note*. SCP (Senior Companion Program). Egos refer to survey participants whereas alters refer to nominated friends. The “Frequency of interaction with the alter in the past month” refers to the frequency of interaction between an ego and one specific alter. “Meetings” refer to the frequency of interactions (e.g., inperson, phone) between ego and alter in the past month. When participants did not provide information on the number of times they met with an alter, their frequencies of the meeting were imputed as 0. Fourteen values were imputed.

**Figure:**
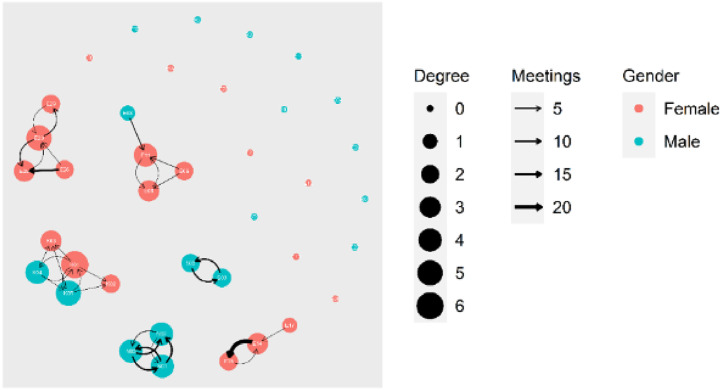


**Figure:**
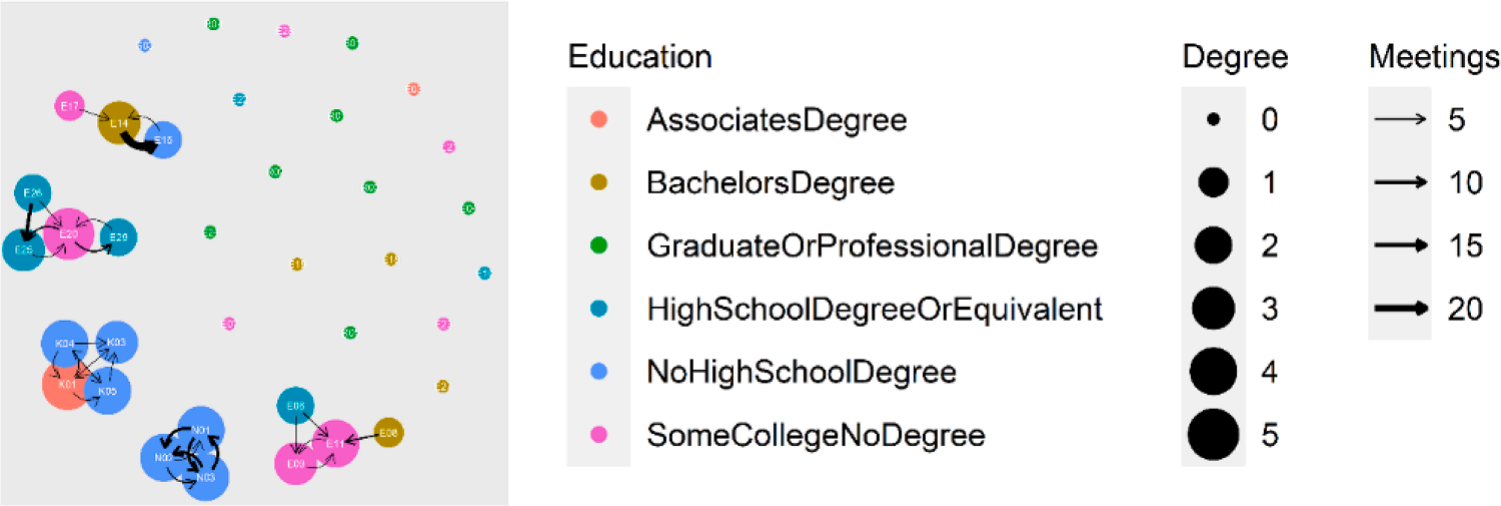


**Figure:**
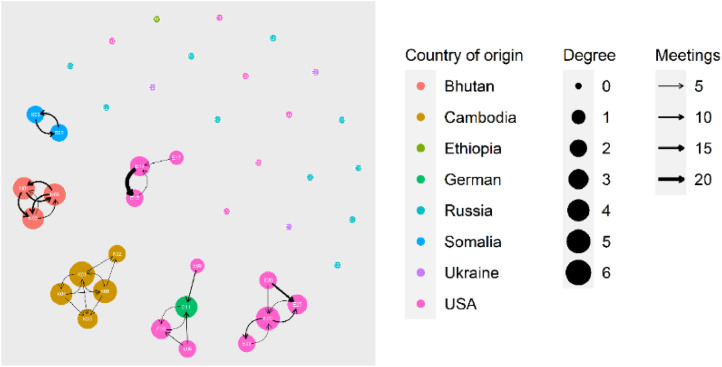


**Figure:**
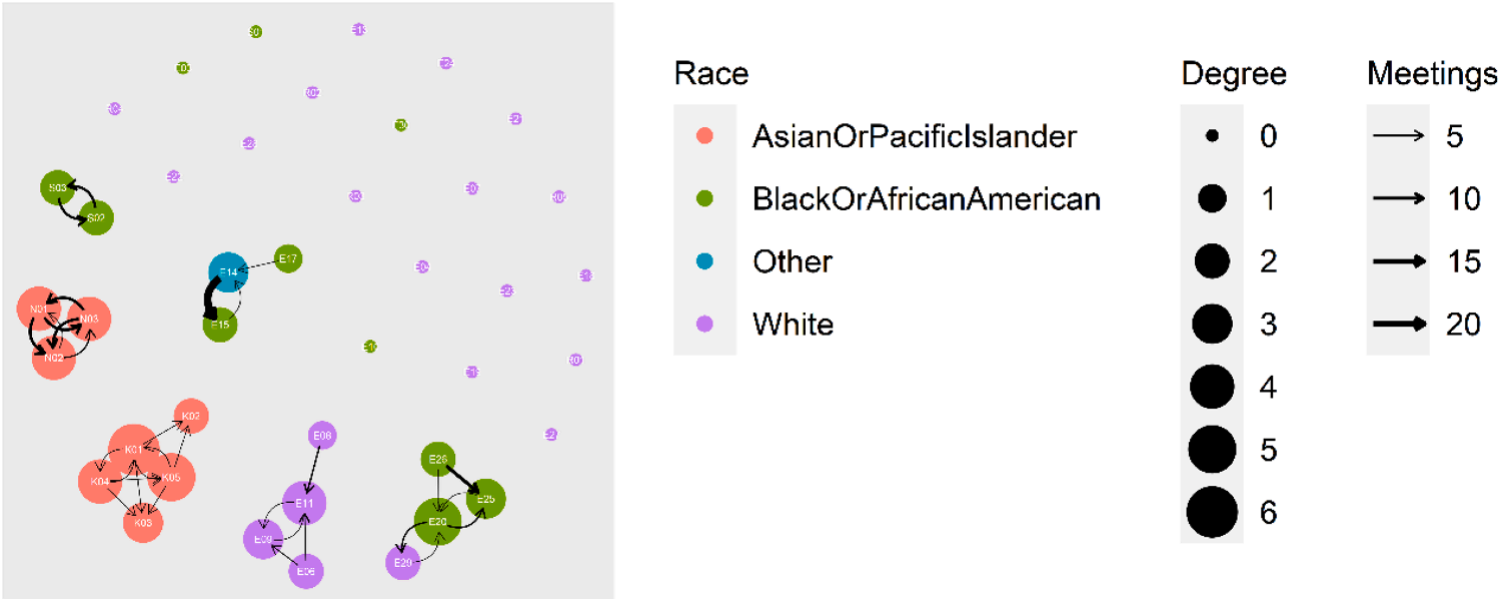


**Figure:**
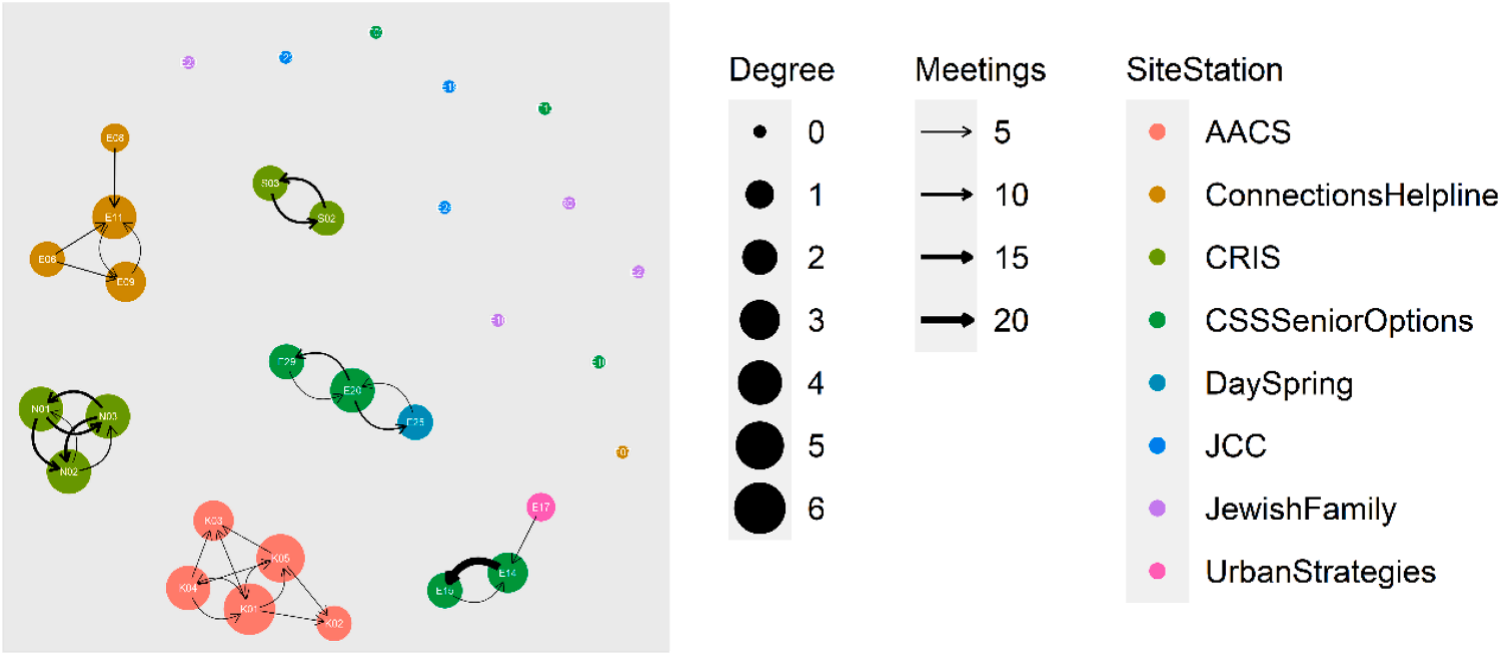
*Note*. The degree of each node in the network graph reflected the total degree of each ego (Freeman, 1979). Meeting refers to the frequency of meetings between egos and alters. AACS refers to Asian American Community Services. JCC refers to Jewish Community Center. CRIS refers to Community Refugee and Immigration Services.

**Figure:**
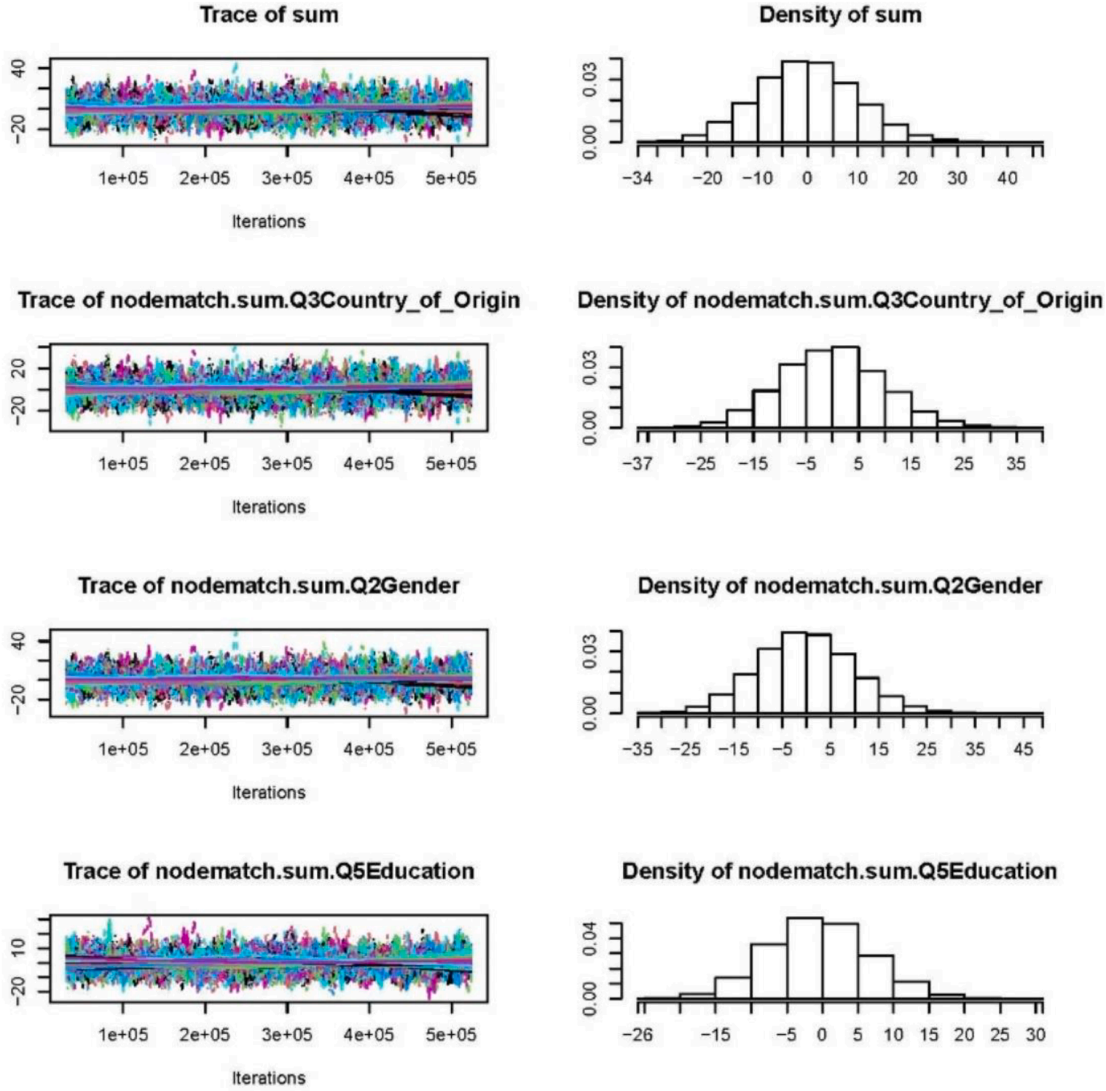

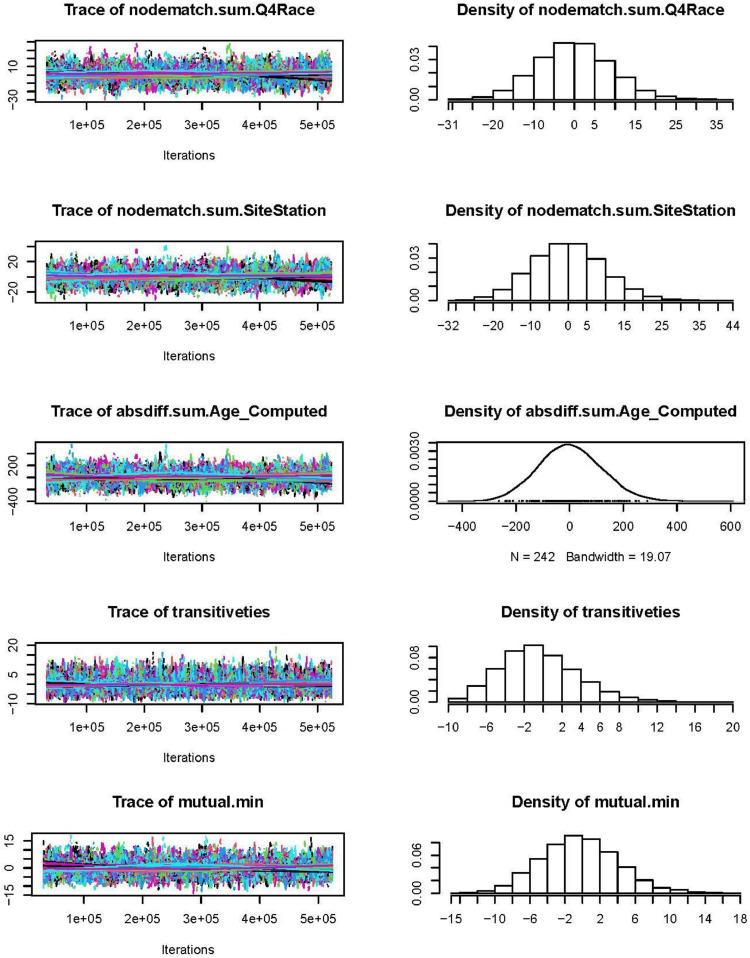


## Appendix II Methodological Details in Exponential Random Graph Models (ERGM)

### Degeneracy in ERGM

As a simulation-based approach, a major challenge with ERGM is its numerical instability, referred to as degeneracy (Cranmer et al., 2020). Degeneracy occurs when the MCMC simulation process moves from completely empty to completely connected networks and is thus unable to maximize the most possible parameter estimates given the observed network. When a model is degenerate, the parameter estimates are not trustworthy. Degeneracy is often caused by poor model specification, such as forcefully fitting network structures that are highly unlikely in the observed network (Cranmer et al., 2020). Fortunately, degeneracy can be easily detected through model fit analysis. A degenerate model has a very poor fit (Cranmer et al., 2020).

### Count ERGM

Building on the above approach of estimating ERGMs for binary edge existence, count ERGM was implemented for this weighted network (Krivitsky, 2012). Despite similarities in assumptions and specification with binary ERGM estimating tie existence versus non-existence, count ERGM required the specification of a reference measure, a probability distribution that provides initial constraints for dyad distribution and network parameters. The Poisson distribution was selected for this count ERGM, based on the large dispersion of the frequency of meetings across ties, as shown in Table 1. In these conditions, the count ERGM was expressed by the following probability function (Krivitsky, 2012):

(1) $$P\left(y\right)=P_{\theta ;g,h}\left(y|x\right)=\frac{h\left(y\right)\exp\left(\theta \cdot g\left(y;x\right)\right)}{\kappa_{g,h}\left(\theta ;x\right)},y\in Y,\;\theta \in R^{p},\;g:Y\to R^{p},\;h:Y\to \left[0,\infty \right),$$

where P(y) was the probability of observing network y from among all networks that could be possibly constructed on the given set of nodes. The set of all these networks was called Y. P(y) depended on a vector g consisting of p network statistics, a vector θ consisting of p fitted parameters, a function *h* (see below), and possibly some node data x. We expressed this via the form $P\left(y\right)=P_{\theta ;g,h}\left(y|x\right)$, meaning that P(y) was the probability of observing network y given node data x, based on our selection of functions g,h, and fitted parameters θ.

Vector θ had p components which were real numbers: $\theta \in R^{p}$. Vector g had p components as well and it was a map from space Y to a space of real numbers: $g:Y\to R^{p}$. Vector g was a function g(y;x) consisting of network statistics on y that could include, for example, the count of transitive ties or the count of reciprocal pairs shown in Appendix I. Function g(y;x) could also include node data x such as homophily based on race, gender, or recruitment site.

The exponential argument to P(y) was the scalar product θ·g(y;x), a real number. The exponential term exp(θ·g(y;x)) was thus a positive value. Function h(y) was chosen in a way that made it positively valued, and was used to specify the reference measure, a baseline probability distribution for the dyad values such as the number of meetings (Krivitsky, 2012). The reference measure and the exponential term worked together as the probability weight h(y)exp(θ·g(y;x)). The weight was normalized using an appropriate normalization expression $\kappa_{g,h}\left(\theta ;x\right)$ as a denominator in Eq. (1). To be exact, $\kappa_{g,h}\;$was given by

(2) $$\kappa_{g,h}\left(\theta ;x\right)=\sum_{y^{\prime }\in Y}h\left(y^{\prime }\right)\exp\left(\theta \cdot g\left(y^{\prime };x\right)\right).$$

Further details on the construction of count ERGMs can be found in (Krivitsky, 2012). The algorithms that fitted the parameters θ and examined their significance were implemented in the *ergm.count* package for the R statistical environment. Model fit was assessed to rule out degeneracy (Cranmer et al., 2020). The variance inflation factor (VIF) among independent variables is produced by the *vif.ergm* function from the R package *ergMargins* to rule out multicollinearity (Duxbury, 2021).

## Appendix VIII Network Graph by Recruitment Sites

*Note*. The degree of each node in the network graph reflected the total degree of each ego (Freeman, 1979). Meeting refers to the frequency of meetings between egos and alters. AACS refers to Asian American Community Services. JCC refers to Jewish Community Center. CRIS refers to Community Refugee and Immigration Services.

## Appendix J Supplementary materials

Image, application 1

Image, application 2
